# Evolution of patients with chronic pain undergoing standard treatment: a prospective longitudinal follow-up study

**DOI:** 10.1016/j.bjane.2025.844700

**Published:** 2025-11-01

**Authors:** Camila Cavalcante Castro, Sandro Max C. Silva, Martha Moreira C. Castro, Durval Campos Kraychete, Carla Daltro

**Affiliations:** a Universidade Federal da Bahia, Postgraduate Program in Medicine and Public Health, Salvador, BA, Brazil; b Universidade Federal da Bahia, Postgraduate Program in Integrative Processes of Organs and Systems, Salvador, BA, Brazil; c Universidade Federal da Bahia, Multidisciplinary Institute of Rehabilitation and Health, Salvador, BA, Brazil

**Keywords:** Anxiety, Chronic pain, Clinical evolution, Depression, Pain measurement

## Abstract

**Introduction:**

Chronic pain greatly affects quality of life and, consequently, impacts the psychological state, a condition that needs to be addressed. A 30% reduction in pain intensity is clinically significant. The objective of this study was to describe the clinical and psychological aspects of individuals with chronic pain undergoing standard treatment.

**Methods:**

Descriptive longitudinal study involving individuals with chronic pain undergoing treatment at the Pain Outpatient Clinic of the Federal University of Bahia, in Salvador, Bahia, between June 2016 and December 2017. The variables studied were pain intensity, quality of life, sleep disorders, stress level, and the presence of anxiety and depression symptoms. Descriptive statistics were performed, and Student's t-test, and Fisher's Chi-Square test were used to compare the groups.

**Results:**

We studied 134 individuals with a mean (standard deviation) age of 50 (10) years, 89.6% of whom were female. There was an improvement in quality of life and sleep, anxiety and depressive symptoms, and 58.2% of patients showed a 30% reduction in pain intensity. Among the factors associated with pain reduction, having a partner was a significant factor (73.7% vs. 52.1%; p = 0.030). However, symptoms of anxiety (81.6% vs. 75.0%; p = 0.436), symptoms of depression (63.2% vs. 58.3%; p = 0.718), and stress (92.1% vs. 87.5%; p = 0.846) were not associated with pain reduction.

**Conclusion:**

This study suggests that multidisciplinary treatment can reduce pain intensity in chronically affected patients, as most patients exhibited a clinically significant response, accompanied by global improvement.

## Introduction

In 2020, a task force comprised of members of the International Association for the Study of Pain (IASP), and the World Health Organization (WHO) reviewed the pathophysiological and research concepts of pain. They added that pain is an individual perception, the report of which must be respected and is directly associated to each person’s life experiences.^1^ It is estimated that, worldwide, around 60 million people suffer from chronic pain, corresponding to 10% of the global population, with lower back pain being the most prevalent location, followed by headaches. In contrast, a systematic review revealed that approximately 45.6% of the Brazilian population suffers from the same condition, representing around 95 million people, and this condition is more prevalent in the Central-West region.^2^ Pain is the primary complaint that explains the frequency with which these individuals seek health services.^3^

Pain influences several aspects of a person’s life and contributes to a decline in quality of life.^4^ A systematic review analyzed 10 double-blind studies with a total of 2,724 individuals with chronic pain. It concluded that a reduction of approximately two points on the numerical pain scale, or a 30% decrease, represents a clinically significant improvement.^5^ This reduction is more effective when a planned multidisciplinary approach is adopted.

Considering the impact of chronic pain on a person’s life and the improvement in pain intensity with multidisciplinary treatment, this study aimed to describe the clinical and psychological evolution of subjects with chronic pain treated by a multidisciplinary team at a specialized referral center in the Unified Health System (SUS). The central hypothesis is that treatment in a specialized center leads to improvement in pain intensity. Quantitative analysis of data obtained in a survey with a representative sample was used to test this hypothesis.

## Methods

### Type of research

This descriptive longitudinal study was conducted at the Pain Outpatient Clinic of the University Hospital of the Federal University of Bahia, Salvador, Bahia, Brazil. It included individuals with chronic pain who were undergoing standard treatment according to the WHO analgesic ladder.^6^

### Participants and procedures

The subjects were interviewed three times between June 2016 and December 2017: at the initial consultation, and then 3 and 6 months later. At the initial consultation, each subject completed both the sociodemographic questionnaire and the evaluation scales. At the two subsequent consultations, each subject only completed the evaluation scales. Subjects of both sexes aged 18–80 years and regularly enrolled in the outpatient care service, and who were present at all three appointments were included. Those diagnosed with pain of oncological origin and who had difficulty understanding the study were excluded.

### Research development

**Figure 1:**
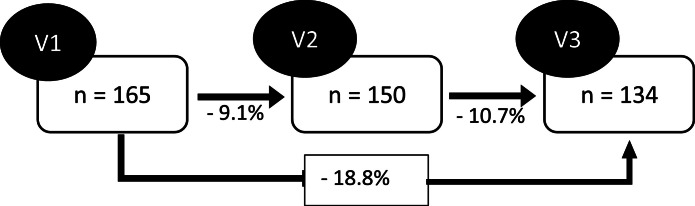
The number of participants at each time point of the study ‒ V1 (initial), V2 (after 3 months), and V3 (after 6 months) ‒ and the percentage loss at each time. At each time, all instruments were applied to subjects with pain treated at Pain Outpatient Clinic of the University Hospital of the Federal University of Bahia. Note: Only the 134 patients who had data from all three visits were included in the sample.

Once a subject was enrolled in the service, during the initial consultation, the unit’s attending physician confirmed a previous diagnosis of chronic pain in the subject’s medical report. The same researcher, a psychologist with a PhD and 25 years of experience in chronic pain care, applied all scales. The research was conducted over 18 months; however, the subjects were included at different times, as shown in Figure 1.

### Instruments

A sociodemographic questionnaire was used to collect the age, sex, marital status, religion, educational background, and employment status of each subject.

A Visual Numeric Scale (VNS) was used to assess pain intensity, ranging from 0 (no pain) to 10 (unbearable pain). A 30% reduction in pain intensity was considered to indicate a clinically significant improvement.^7^

The Short Form-36 (SF-36) was used to assess quality of life. It contains eight domains: functional capacity assesses physical capacity; physical aspect assesses physical limitations; pain assesses the presence of pain and interference in activities of daily living; general health assesses overall health; vitality assesses energy level and fatigue; social aspect assesses integration in social activities; emotional aspect analyzes the impact of psychological aspects on the patient’s well-being; and mental health assesses symptoms and psychological well-being. The scale ranges from 0 to 100, with 0 indicating the worst health status and 100 indicating the best health status. Given that it is a subjective assessment, it has no cut-off point.^8^

The Mini-Sleep Questionnaire (MSQ) assesses the presence of sleep disorders. The score is categorized as follows: 10–24 points indicate “good sleep”; 25–27 points indicate “mild disorder”; 28–30 points indicate “moderate disorder”; and > 30 points indicate “severe disorder”.^9^

The Pittsburgh Sleep Quality Index (PSQI) is a scale that measures sleep quality. The maximum total score is 21 points. The higher the score, the worse the sleep quality.^10^

The Hospital Anxiety and Depression Scale (HAD) assess the presence of anxiety and depression symptoms. It has a cut-off point of 8 for anxiety and 9 for depression.^11^

Lipp’s Stress Symptom Inventory for Adults (LSSI) is an instrument comprising 37 items, divided into three tables that refer to the phases of stress. The first table pertains to physical or psychological symptoms experienced in the last 24 hours, the second to those experienced in the previous week, and the third to those experienced in the previous month.^12^

### Statistical analysis

SPSS Statistics version 17.0 was used for data analysis. Quantitative variables are expressed as mean and Standard Deviation (SD), and categorical variables are expressed as absolute and relative frequency. Descriptive statistics, normality graphs (histogram, boxplot, and Q–Q plots), and the Shapiro-Wilk test were used to assess the normality of the variables. The variables, expressed as scores and evaluated by the scales, were considered ordinal and are presented as medians and interquartile ranges.

Patients were initially divided into two groups: those who showed any improvement in pain intensity (one or more VNS scores) and those who showed no improvement or worsened. The scales related to quality of life, sleep, stress, and anxiety and depression symptoms were then compared at the initial (V1) and final (V3) visits. The McNemar and Wilcoxon tests were used to compare categorical and ordinal variables, respectively.

Considering that a 30% reduction in pain intensity is a good clinical response to treatment for this condition, and to investigate the factors associated with this improvement, patients were again divided into two groups based on this characteristic: those who achieved less than 30% improvement or no improvement, and the other group with an improvement in the VNS score greater than or equal to 30% compared to baseline (V1). Student's *t*-test, Pearson's Chi-Square test, and Fisher's exact test were used to compare these groups. Binary logistic regression was performed to investigate the effect of confounding variables associated with pain intensity improvement. The following variables were included in the model: gender, marital status, anxiety symptoms, depression and stress symptoms (independent variables), and pain intensity reduction (dependent variable). The reasons for including variables in the model were: plausibility of interference in the association and p-value < 0.05 in the bivariate analysis. A p-value < 0.05 was considered to be statistically significant.

## Results

A total of 165 individuals were selected, but only those who attended all three moments comprised the sample. Thus, 134 were included in the sample, representing a loss of 18.8% due to difficulty in contacting, treatment abandonment, change of address, or other reasons. The sociodemographic characteristics observed in the group lost to follow-up are similar to the study sample, with a mean age of 49.4 (10.6), all female, the majority had (60%) and religion (85%), and pain intensity had a median of 7 (6‒8). The mean (SD) age was 50 (10) years. Most of the subjects were female (89.6%), had a partner (58.2%), were of mixed race (51.5%), were unemployed (63.4%), and declared a religion (94.8%) (Table 1). In addition, 59.7% reported that they did not perform physical activity regularly, 7.5% consumed alcohol, and 18.7% smoked. Regarding the pain pattern, 51.5% reported that there was no specific time for the pain to become more intense; however, 25.4% reported that the pain worsened at night.

**Table 1:** Sociodemographic characteristics of 134 patients with chronic pain treated at the Pain Outpatient Clinic of C-HUPES/UFBA, Salvador‒Bahia.

Variables	Results
Age in complete years^a^	50 (10)
Female Sex	120 (89.6%)
Marital Status	
With partner	78 (58.2%)
Without partner	56 (41.8%)
Religion	127 (94.8%)
Education	
No education	2 (1.5%)
Elementary school complete and incomplete	50 (37.3%)
High school complete and incomplete	71 (53%)
High school complete and incomplete	11 (8.2%)
Ethnicity	
Mulatto/Mixed race	69 (51.5%)
Black	38 (28.4%)
White	27 (20.1%)
Employment Status	
Employed	35 (26.1%)
Unemployed	85 (63.4%)
Retired	14 (10.4%)
Pain Intensity^a^	7 (6 - 8)

a Values expressed as mean and standard deviation.

For comparison purposes, the subjects were divided into two groups based on the evolution of pain: those who showed improvement in pain, representing 58.2% of the subjects, and those who remained with an unchanged or worsening pain level, representing 41.8% of the subjects. The group that showed an improvement in pain also showed improvement in all domains of the SF-36, depressive and anxiety symptoms, stress level, and sleep pattern. However, the group that showed no change or worse pain improved in only one domain of the SF-36 and the sleep pattern (Table 2).

**Table 2:** Evolution of 134 patients with chronic pain before and after six months of follow-up divided into groups in which the pain improved or worsened/remained unchanged at the Pain Outpatient Clinic of C-HUPES/UFBA, Salvador‒Bahia, 2016‒2017.

Variables	Worsened/Unchanged 56 (41.8%)		p	Improved 78 (58.2%)		**p**
	V1	V3		V1	V3	
Anxiety Symptoms	39 (70%)	45 (80%)	0.180	64 (82%)	52 (67%)	0.038
Depressive Symptoms	28 (50%)	30 (54%)	0.508	52 (67%)	30 (39%)	0.001
Stress	46 (82%)	45 (80%)	1.000	73 (94%)	58 (74%)	0.001
Quality of Life						
Functional Capacity	30 (15–45)	30 (20–45)	0.331	25 (15–35)	42 (32–52)	< 0.001
Physical Limitations	0 (0–25)	23 (0 – 35)	0.021	0 (0–0)	42 (0–54)	< 0.001
Pain	22 (12–35)	24 (22–41)	0.460	22 (12–31)	42 (34–53)	< 0.001
General Health	34 (23–51)	37 (25–52)	0.929	35 (23–50)	48 (36–58)	< 0.001
Vitality	27 (15–59)	35 (25–56)	0.252	26 (15–35)	55 (40–67)	< 0.001
Social Aspects	42 (13–57)	38 (25–58)	0.929	38 (25–50)	50 (39–63)	< 0.001
Emotional Limitations	33 (0–68)	33 (0–51)	0.320	0 (0–89)	50 (26–69)	0.008
Mental Health	42 (24–68)	37 (20–52)	0.223	38 (28–65)	46 (37–66)	0.029
Sleep						
Quality	13 (10–17)	12 (6–14)	0.001	14 (10–17)	8 (5–11)	< 0.001
Disorders	44 (32–49)	38 (30–48)	0.016	46 (41–53)	31 (28–34)	< 0.001
Pain Intensity	6 (5–7)	6 (5–8)	<0.001	8 (7–9)	5 (5–6)	< 0.001

Note: McNemar and Wilcoxon tests were used.

Regarding the factors associated with clinically significant pain improvement (i.e., ≥ 30% improvement during treatment at the specialized center), only having a partner was associated with a reduction in pain intensity (Table 3).

**Table 3:** Factors associated with pain reduction in 134 patients with pain being treated at the Pain Outpatient Clinic of C-HUPES/UFBA, Salvador‒Bahia.

Variables	Pain Reduction		p	OR (95% CI)
	≥ 30%	< 30%		
	38 (28.4%)	96 (71.6%)		
Age	51 (10)	50 (10)	0.757	‒
Sex			0.220	1.807 (0.545 ‒ 5.990)
Female	32 (84.2%)	88 (91.7%)		
Male	6 (15.8%)	8 (8.3%)		
With partner	28 (73.7%)	50 (52.1%)	**0.022**	**2.533 (1.094 – 5.869)**
Anxiety Symptoms	31 (81.6%)	72 (75%)	0.416	0.602 (0.168 – 2.157)
Depressive Symptoms	24 (63.2%)	56 (58.3%)	0.608	1.216 (0.422 – 3.505)
Stress (ISSL)	35 (92.1%)	84 (87.5%)	0.555	0.869 (0.211 – 3.586)

Note 1: The *t*-test, Pearson's Chi-Square test and Fisher's exact test were used.

Note 2: Variables were included in the model: gender, marital status, anxiety symptoms, depression and stress symptoms (independent variables), and pain intensity reduction (dependent variable).

## Discussion

In this study, most subjects treated for chronic pain who experienced an improvement in their pain intensity also showed an improvement in their sleep patterns, stress, anxiety, and depression symptoms, and all domains of quality of life. The group that showed unchanged or worsened pain only improved in terms of sleep and in only one domain of the quality-of-life scale. In addition, having a partner was associated with a greater reduction in pain intensity, perhaps due to better therapeutic adherence (i.e., having another person to dispense medications) as well as financial and emotional support.^13^ This, however, is a hypothesis generated in this study that should be interpreted with caution.

The negative impact of chronic pain on quality of life is well known. Our findings support the association between reduced pain intensity and an overall improvement in quality of life. The specific improvement in the physical aspect’s domain in subjects with no change in pain or worsened pain intensity may be due to the subject’s ability to reframe pain by adapting to these limitations, in addition to the multidisciplinary treatment that improves mobility.^14^ A 2022 study involving 379 participants demonstrated that the perception of quality of life varies according to the intensity of pain.^15^

Pain is one of the triggers of stress, generating physiological responses that release catecholamines and increase cortisol production. If prolonged, this situation impairs daily activities and physiological cycles.^16^ These changes may be associated with genetic factors that make susceptible subjects more sensitive to the effects of catecholamines and, consequently, increase pain. The OPPERA study evaluated the factors associated with the development of orofacial pain. It concluded that stress only acts as an additive risk factor for increased pain in individuals without preexisting psychological symptoms.^17^

Chronic pain leads to a pattern of depressive and anxious responses with symptoms such as low mood and changes in sleep patterns.^18^ The greater the intensity of pain and the number of painful points, the greater the magnitude of these symptoms tends to be.^19^ In our study, the group that showed an improvement in pain intensity also showed a reduction in the number of subjects with anxious and depressive symptoms, corroborating the aforementioned association.

Chronic pain treatment guidelines from around the world agree that interdisciplinary intervention is necessary, with pharmacological treatment being one of the pillars.^20^ During this study, the subjects underwent psychological evaluation with a cognitive-behavioral approach. This intervention has been shown to improve pain and might explain the improvement in the pattern of depressive and anxious responses presented by these subjects.^21^

Changes in sleep patterns are risk factors for the exacerbation and chronicity of pain. On the other hand, impaired sleep quality exacerbates pain, highlighting a bidirectional connection between sleep and pain, which is due to modifications in the circadian cycle and modulation of neurotransmitters associated with this process.^22^ The improved sleep patterns of the subjects may have been due to the use of adjuvant drugs such as antidepressants. Although antidepressants are not commonly used at therapeutic doses to treat sleep disorders, their most common adverse effect is drowsiness. Moreover, according to the WHO analgesic ladder, regardless of the level of pain, the use of antidepressants is recommended.^23^

The intensity of pain and its interference with the life of a subject with chronic pain are determinants of the severity of the disease.^24^ Improvement in this parameter reflects a clinical improvement for these subjects. When treating patients with chronic pain, any clinical improvement is important; hence, according to the literature, a 30% reduction in pain score relative to the initial score is considered clinically significant.^25^ This may be attributed to the multidisciplinary treatment of the subject.

Although there was no control over medication use in this study, the improvement observed in pain intensity, regardless of the pathophysiological mechanism and etiological diagnosis, may be attributed to the fact that the subjects were part of an outpatient clinic specializing in chronic pain and had access to a multidisciplinary team.

Other authors have also observed the link between clinical improvement in the underlying condition and marital status. Vance et al.^25^ conducted a randomized clinical trial in 2021 with 301 patients in the United States to evaluate the response to Transcutaneous Electrical Nerve Stimulation (TENS) in women with fibromyalgia. They found that married women responded better to the treatment. The authors attributed this to better adherence to the therapeutic protocol; we also speculate that this group had greater social support, which contributed to the positive outcome.

This study had some limitations. First, we were unable to confirm the etiology of the pain, as the subjects entered the service with a diagnosis provided by the attending physician. Second, although the subjects received standard treatment, it was not possible to determine the frequency of consultations, the interventions performed, or adherence to medication therapy, as these were individualized. Another aspect is the possibility of unmeasured confounding factors, as well as losses during the longitudinal study (18.8%). Finally, the convenience sample may compromise the sample size and, consequently, the power of the study.

On the other hand, we consider possible biases, such as selection and confounding, to be unlikely. First, all patients were invited and accepted participation, which made selection bias possible; second, the second bias was minimized by controlling for potential confounding variables using binary logistic regression.

Generalization of data should be done with caution. Given that the patients studied were followed up at an outpatient clinic of the Unified Health System, data/results should only be generalized to similar services.

In future studies, the specific drug therapy should be determined, and there should be greater control of therapeutic adherence to establish assertive longitudinal study criteria that will improve the quality of the service provided.

## Conclusion

In this observational study, approximately half of the subjects with chronic pain reported a clinically significant reduction in pain after standard treatment. The subjects also showed improvements in sleep quality and emotional well-being. However, given the absence of a control group and the observational design, these findings should be interpreted with caution.

The evolution of pain intensity is associated with the presence of anxiety and depression symptoms, stress, sleep patterns, and quality of life. Although the subjects who reported improvement in pain intensity also showed improvement in the other parameters studied, it is not possible to establish whether improvements in the psychological status and sleep improve pain or vice versa.

## Ethical aspects

This study was conducted in accordance with the principles of the Declaration of Helsinki. It was approved by the Research Ethics Committee of Professor Edgard Santos University Hospital (opinion nº 1.446.343 and CAAE nº 4990615.8.0000.0049).

## Data availability statement

The data supporting the findings of this study are available upon reasonable request to the corresponding author [CC]. The data are not publicly available due to ethical constraints, such as containing information that could compromise the privacy of research participants.

## Conflicts of interest

The authors declare no conflicts of interest.

## Appendix Supplementary materials

Image, application 1
