# Supplementary material for: Evolution of patients with chronic pain undergoing standard treatment: a prospective longitudinal follow-up study
Source: Braz J Anesthesiol. 2025 Nov 1;76(1):844700. doi: 10.1016/j.bjane.2025.844700 (PMC12765431; doi:10.1016/j.bjane.2025.844700)
Supplement: Supplementary file 1 [file mmc1.pdf]

PARECER CONSUBSTANCIADO DO CEP

DADOS DO PROJETO DE PESQUISA

Título da Pesquisa: DOR CRÔNICA: AVALIAÇÃO E TRATAMENTO CLINICO
Pesquisador: Martha Moreira Cavalcante Castro
Área Temática:
Versão: 3
CAAE: 49909615.8.0000.0049
Instituição Proponente: Hospital Universitário Prof. Edgard Santos-UFBA
Patrocinador Principal: Financiamento Próprio

DADOS DO PARECER

Número do Parecer: 1.446.343

Apresentação do Projeto:
Trata-se de 3a versão de protocolo descrito no parecer Número 1.380.826 de 23/12/2015, identificado com pendências, as quais foram respondidas por meio da adição de uma nova versão do TCLE, em arquivo intitulado "Tcle_ajustado.pdf", datado de 03/03/2016.

Objetivo da Pesquisa:
Vide parecer Número 1.380.826 de 23/12/2015.

Avaliação dos Riscos e Benefícios:
Vide parecer Número 1.380.826 de 23/12/2015.

Comentários e Considerações sobre a Pesquisa:
Vide parecer Número 1.380.826 de 23/12/2015.

Considerações sobre os Termos de apresentação obrigatória:
Vide parecer Número 1.380.826 de 23/12/2015.

Recomendações:
Vide parecer Número 1.380.826 de 23/12/2015.

Conclusões ou Pendências e Lista de Inadequações:
As pendências abaixo elencadas foram atendidas nesta versão.

6) De acordo com o item IV.5.d da resolução 466/2012, o TCLE deve:

b) "devendo as páginas de assinaturas estar na mesma folha". SOLICITA-SE ADEQUAÇÃO; ESSA PENDÊNCIA SE MANTEM UMA VEZ QUE O ARQUIVO INTITULADO "TCLE_AJUSTADO.DOC" APRESENTA DUAS PAGINAS DE ASSINATURAS, SENDO A DO PARTICIPANTE EM UMA E A DO PESQUISADOR EM OUTRA.
PENDÊNCIA RESOLVIDA;

c) "Em ambas as vias deverão constar o endereço e contato telefônico ou outro, dos responsáveis pela pesquisa e do CEP local e da CONEP, quando pertinente". SOLICITA-SE ADEQUAÇÃO; ESSA PENDÊNCIA SE MANTEM UMA VEZ QUE O ARQUIVO INTITULADO "TCLE_AJUSTADO.DOC" NÃO APRESENTA EM NENHUM LOCAL OS CONTATOS DO CEP LOCAL. PENDÊNCIA RESOLVIDA.

Considerações Finais a critério do CEP:

O participante da pesquisa tem a liberdade de recusar-se a participar ou de retirar seu consentimento em qualquer fase da pesquisa, sem penalização alguma e sem prejuízo ao seu cuidado (Res. CNS 466/12) e deve receber uma cópia do Termo de Consentimento Livre e Esclarecido, na íntegra, por ele assinado.

O pesquisador deve desenvolver a pesquisa conforme delineada no protocolo aprovado e descontinuar o estudo somente após análise das razões da descontinuidade pelo CEP que o aprovou, aguardando seu parecer, exceto quando perceber risco ou dano não previsto ao sujeito participante ou quando constatar a superioridade de regime oferecido a um dos grupos da pesquisa que requeiram ação imediata.

O CEP deve ser informado de todos os efeitos adversos ou fatos relevantes que alterem o curso normal do estudo. É papel do pesquisador assegurar medidas imediatas adequadas frente a evento adverso grave ocorrido (mesmo que tenha sido em outro centro) e enviar notificação ao CEP e à Agência Nacional de Vigilância Sanitária – ANVISA – junto com seu posicionamento.

Eventuais modificações ou emendas ao protocolo devem ser apresentadas ao CEP de forma clara e sucinta, identificando a parte do protocolo a ser modificada e suas justificativas.

Relatórios parciais e final devem ser apresentados ao CEP, inicialmente em ______/_____/_____ e ao

término do estudo.

Situação: Projeto Aprovado.

Este parecer foi elaborado baseado nos documentos abaixo relacionados:

Tipo Documento	Arquivo	Postagem	Autor	Situação
Informações Básicas do Projeto	PB_INFORMAÇÕES_BÁSICAS_DO_PROJETO_461692.pdf	03/03/2016 22:51:12		Aceito
TCLE / Termos de Assentimento / Justificativa de Ausência	TCLE_Ajustado.pdf	03/03/2016 22:50:41	Martha Moreira Cavalcante Castro	Aceito
TCLE / Termos de Assentimento / Justificativa de Ausência	TCLE_Ajustado.doc	21/01/2016 00:16:33	Martha Moreira Cavalcante Castro	Aceito
Outros	RESPOSTA_AO_PARECER_CONSUBSTANCIADO_DO_CEP.docx	21/01/2016 00:16:04	Martha Moreira Cavalcante Castro	Aceito
Outros	ANAMNESE_GRUPO_DOR.doc	21/01/2016 00:15:24	Martha Moreira Cavalcante Castro	Aceito
Outros	escala_sono2.jpg	21/01/2016 00:14:57	Martha Moreira Cavalcante Castro	Aceito
Outros	escala_sono1.jpg	21/01/2016 00:14:04	Martha Moreira Cavalcante Castro	Aceito
Outros	ESCALA_SF36.doc	21/01/2016 00:13:30	Martha Moreira Cavalcante Castro	Aceito
Outros	ESCALA_HAD.doc	21/01/2016 00:12:32	Martha Moreira Cavalcante Castro	Aceito
Outros	DECLARACAO_DE_CONFIDENCIALIDADE.jpg	21/01/2016 00:12:12	Martha Moreira Cavalcante Castro	Aceito
Outros	CARTA_DE_ENCAMINHAMENTO.jpg	21/01/2016 00:11:30	Martha Moreira Cavalcante Castro	Aceito
Outros	CARTA_DE_ANUENCIA.jpg	21/01/2016 00:11:07	Martha Moreira Cavalcante Castro	Aceito
Projeto Detalhado / Brochura Investigador	Projeto_ajustado_coorte_MARTHA.docx	21/01/2016 00:08:50	Martha Moreira Cavalcante Castro	Aceito
Outros	LIPP_6.jpg	21/01/2016 00:08:02	Martha Moreira Cavalcante Castro	Aceito
Outros	LIPP_5.jpg	21/01/2016 00:07:33	Martha Moreira Cavalcante Castro	Aceito
Outros	LIPP_4.jpg	21/01/2016 00:07:15	Martha Moreira Cavalcante Castro	Aceito

Endereço: Rua Augusto Viana, s/nº - 1º Andar
Bairro: Canela **CEP:** 40.110-060
UF: BA **Município:** SALVADOR
Telefone: (71)3283-8043 **Fax:** (71)3283-8140 **E-mail:** cep.hupes@gmail.com

Outros	LIPP_3.jpg	21/01/2016 00:05:21	Martha Moreira Cavalcante Castro	Aceito
Outros	LIPP_2.jpg	21/01/2016 00:05:00	Martha Moreira Cavalcante Castro	Aceito
Outros	LIPP_1.jpg	21/01/2016 00:02:39	Martha Moreira Cavalcante Castro	Aceito
Outros	EVA.jpg	20/01/2016 23:50:23	Martha Moreira Cavalcante Castro	Aceito
Folha de Rosto	FolhadeRostoassinada.pdf	07/10/2015 17:01:03	Martha Moreira Cavalcante Castro	Aceito
Outros	termo de compromisso do investigador.pdf	20/07/2015 20:23:31		Aceito
TCLE / Termos de Assentimento / Justificativa de Ausência	TCLE 5 .pdf	20/07/2015 20:20:16		Aceito
TCLE / Termos de Assentimento / Justificativa de Ausência	TCLE 4 .pdf	20/07/2015 20:19:42		Aceito
TCLE / Termos de Assentimento / Justificativa de Ausência	TCLE 3 .pdf	20/07/2015 20:19:06		Aceito
TCLE / Termos de Assentimento / Justificativa de Ausência	TCLE 2.pdf	20/07/2015 20:18:34		Aceito
TCLE / Termos de Assentimento / Justificativa de Ausência	TCLE 1.pdf	20/07/2015 20:18:06		Aceito
Outros	orçamento financeiro.pdf	20/07/2015 20:16:53		Aceito
Outros	equipe detalhada 2.pdf	20/07/2015 14:03:58		Aceito
Outros	equipe detalhada 1.pdf	20/07/2015 14:03:21		Aceito
Outros	encaminhamento projeto CEP.pdf	20/07/2015 14:02:35		Aceito
Outros	declaração de confidencialidade do sujeito.pdf	20/07/2015 14:02:08		Aceito
Outros	cronograma.pdf	20/07/2015 14:01:38		Aceito
Outros	Carta de anuencia .pdf	20/07/2015 14:00:58		Aceito
Projeto Detalhado	PROJETAO MARTHA concluido.docx	20/07/2015		Aceito

/ Brochura Investigador	PROJETAO MARTHA concluido.docx	14:00:11		Aceito

Situação do Parecer:
Aprovado

Necessita Apreciação da CONEP:
Não

SALVADOR, 10 de Março de 2016

Assinado por:
NEY CRISTIAN AMARAL BOA SORTE
(Coordenador)

Endereço: Rua Augusto Viana, s/nº - 1º Andar
Bairro: Canela **CEP:** 40.110-060
UF: BA **Município:** SALVADOR
Telefone: (71)3283-8043 **Fax:** (71)3283-8140 **E-mail:** cep.hupes@gmail.com
